# Individual patterns and synchrony of heart rate variability in adolescent patients with borderline personality psychopathology and their mothers: a case–control study

**DOI:** 10.1186/s40479-025-00289-0

**Published:** 2025-04-09

**Authors:** Katharina Williams, Anna Fuchs, Jana Kuehn, Leonie Fleck, Stefan Lerch, Marialuisa Cavelti, Julian Koenig, Michael Kaess

**Affiliations:** 1 Department of Child and Adolescent Psychiatry, Centre for Psychosocial Medicine, University of Heidelberg, Heidelberg, Germany; 2 Institute of Psychology, University of Heidelberg, Heidelberg, Germany; 3 Department of General Psychiatry, Centre for Psychosocial Medicine, University of Heidelberg, Heidelberg, Germany; 4 Department of Public Mental Health, Medical Faculty Mannheim, Central Institute of Mental Health, University of Heidelberg, Heidelberg, Germany; 5 University Hospital of Child and Adolescent Psychiatry and Psychotherapy, University of Bern, Bern, Switzerland; 6 Department of Psychiatry and Psychotherapy for Children and Young Adults, University of Cologne, Cologne, Germany

**Keywords:** HRV, BPD, Adolescents, Mother–child interaction, Physiological synchrony, Behavioral synchrony

## Abstract

**Background:**

In adolescent Borderline Personality Disorder (BPD), interactions with caregivers often provoke dysregulation. Vagally-mediated heart rate variability (HRV), a physiological marker of regulatory capacities, shows alterations in BPD. Studies on individual and dyadic HRV in adolescents with BPD (BPD-A) and their mothers (BPD-M) are lacking. We examined 1) individual resting state -, reactivity- and recovery- HRV, 2) intrapersonal concordance of interactional quality with HRV, 3) mother-adolescent interpersonal HRV-synchrony and 4) the association of interpersonal HRV-synchrony with behavioral synchrony in a case–control design.

**Methods:**

Thirty-eight (sub)syndromal BPD-A and BPD-M were compared to 35 healthy control adolescents and their mothers (HC-A/-M). HRV was assessed during a positive interaction, a stress task and resting before and after interactions (recovery). Behavior during interactions was observed and coded using the “Coding Interactive Behavior”- Manual. Data were analyzed using multilevel modeling.

**Results:**

BPD-A showed a lower resting HRV than HC-A, while no group differences were found for mothers. From resting to positive interaction, BPD-A/BPD-M/HC-M showed a significant increase in HRV; this increase was not significant for HC-A. HRV-reactivity to stress was not significant in either group but influenced by general emotional and behavioral problems within both adolescent samples. Significant intrapersonal concordance of HRV and behavior could only be found for HC-M during the positive interaction (positive association). For BPD-M, a complete disconnect between behavior and HRV was observed. BPD-dyads and dyads lower in behavioral synchrony displayed HRV-synchrony during stress, in HC-dyads and dyads higher in behavioral synchrony during rest after dyadic interactions.

**Conclusions:**

Our study is the first investigating altered HRV-reactivity, behavior-HRV-concordance and HRV-synchrony in adolescents with BPD traits and their mothers, adding new insight to physiological regulation and co-regulation in adolescent BPD pathology. Limitations and implications of these results are discussed.

**Supplementary Information:**

The online version contains supplementary material available at 10.1186/s40479-025-00289-0.

## Background

Individuals with Borderline Personality Disorder (BPD) often experience disturbances in emotion regulation (ER) which can lead to problematic behavior (e.g. self-harm, suicide attempts, substance abuse) and tumultuous interpersonal relationships [1–4]. Understanding how these difficulties in ER develop may be crucial to advance new treatment approaches. Etiological models of BPD suggest that insufficient caregiver-child co-regulatory processes during childhood hinder the development of secure attachment and the ability to self-regulate (e.g. [5, 6]). A time period that is especially relevant for both the development of ER skills and the onset of mental disorders (including BPD) is adolescence [3]. Therefore, studies with an interpersonal focus investigating both behavioral and physiological processes during caregiver-adolescent interactions are needed [7].

### HRV: a marker of individual regulation

A physiological marker commonly associated with emotional and social regulation is vagally-mediated heart rate variability (HRV), i.e. beat-to-beat variability in a sequence of heartbeats [8]. Higher levels of resting HRV indicate higher regulatory capacities [8–10]. Lower resting HRV has been observed in several psychiatric populations—including adult [11] and adolescent BPD patients [12–14] – and has been associated with emotional dysregulation in children and adolescents [15].

Autonomic flexibility, i.e. HRV adjustment in response to environmental demands [8–10], is discussed as a foundation of social effectiveness [9, 16]. While a decrease in HRV (i.e. vagal withdrawal) is suggested to indicate attention and (over)mobilization of resources in response to stressors, HRV-increase (i.e. vagal augmentation) may signal calmness, readiness for social engagement and disengagement from stressors [17–19].

As BPD is often characterized as a disorder of interpersonal functioning [6, 20], studies examining HRV-reactivity in different interpersonal contexts are needed. In healthy individuals, social interactions of negative valence seem to reduce HRV, while neutral or positive interactions may not induce HRV changes [16]. In adolescents with psychopathology, hypo- and hyper-reactivity has been observed. While depressed adolescents did not show HRV-reactivity in response to parent-adolescent interaction in both positive interactions and a conflict discussion task [21, 22], female adolescents with self-injuring behavior displayed hyper-reactivity in response to their mothers’ aversive behavior during a conflict discussion [22]. For individuals with BPD physiological hyper-reactivity expressed through an excessive decrease of HRV has been hypothesized but could not be confirmed by empirical data [23, 24]. Due to a small number of primary studies that have almost exclusively focused on adults, current findings are still to be interpreted with caution [24].

Recovery or the degree of return to resting HRV levels has been neglected in prior research [19, 24]. Similarly, an important but under-researched aspect of regulatory failures in BPD is a slower return to baseline [25]. More studies on adolescent HRV during parent-adolescent interaction including a recovery condition are necessary.

In BPD, where failing parental co-regulation has been discussed as a developmental pathway and parent-adolescent conflicts seem to escalate symptoms and maintain the disorder, parent-adolescent regulation during interaction deserves more research attention [5, 22]. Amole et al. [21] not only reported blunted HRV-reactivity (i.e. no significant changes in HRV) in depressed adolescent daughters but found the same blunted pattern in their depressed mothers, suggesting that there may be altered HRV in parents of mentally ill adolescents. However, there are no studies examining HRV functioning in parents of adolescents with BPD.

In sum, case–control studies focusing on dynamic HRV in both adolescent BPD patients and their parents during actual parent-adolescent interaction are needed. Ideally, these include not only resting conditions but reactivity to and recovery from pleasant and stressful events.

### Intrapersonal concordance of HRV and behavior

Little is known about the relation between behavior and HRV during parent–child interactions, especially during adolescence. It seems to depend on the dyadic context, e.g. whether HRV is measured during a pleasant, collaborative or a stressful task, or whether participants belong to a clinical group or not [9, 10, 22]. Concordance between HRV and behavior during mother–child interactions though has almost exclusively been highlighted in infancy and toddlerhood, with a strong focus on maternal functioning and community samples [26, 27]. A study by Sturge-Apple et al. [28] investigated a preadolescent sample and found parents’ blunted HRV reactivity to be associated with increased parental hostile behavior in a conflict discussion. Studies also suggest that maternal behavior-HRV associations vary when mothers are maltreating their child [29–31]. More data, especially focused on adolescence, is needed to illuminate associations of interactional quality and HRV during parent–child interaction.

### Behavioral and HRV-synchrony in caregiver-child interactions

Behavioral synchrony describes the coordination between a parent’s and child’s nonverbal behavior and communicative signals during social interactions in ways that enhance positivity, reciprocity, and mutual engagement [32]. Parent–child synchrony in HRV is defined as a dynamic, within-dyad coordination of HRV over time that is directly tied to an interpersonal process [33]. HRV synchrony can be positive (increase/decrease of mother’s and child’s HRV at the same timepoint) or negative (increase of mother’s HRV and decrease of child’s HRV at the same timepoint or vice versa). According to the biobehavioral framework put forward by Feldman [34], parents and children adjust their neurobiological and behavioral rhythms over time and daily experience. This dyadic adjustment then sets the stage for the formation of enduring attachment and provides a base for children’s regulatory development [34]. Similarly, Social Baseline Theory [35] postulates that homeostatic coregulation inherent in human nature serves the conservation of regulatory resources and thus provides evolutionary benefit. However, if coregulation goes awry, resources can deplete more rapidly, further disrupting regulation, social affiliation, and skills such as problem solving or attention allocation [6, 36].

HRV-synchrony has been found in parents and children of various developmental stages, yet, the number of studies focusing on adolescents is very limited and findings are mixed [21, 33]. In early childhood, tasks demanding joint attention and simultaneous engagement in a dyad have been found to promote parent–child HRV-synchrony [37, 38]. Some argue that HRV-synchrony can be induced simply by cognitive-emotional processing of a similar stimulus and does not necessarily require actual interaction or shared face-to-face experiences [39]. Importantly, a number of studies suggest that both context and risk status of the dyad conjointly shape presence and nature of synchrony [38]. In the context of maternal and child/adolescent depression, for example, negative synchrony during positive and stressful interactions was observed (as opposed to positive synchrony in healthy dyads; [21, 40]). In the context of preadolescent Posttraumatic Stress Disorder (PTSD; and also increased maternal PTSD symptoms), tighter positive HRV-synchrony was found during a positive interaction in comparison to war-exposed but resilient dyads [41]. There are several indications in research suggesting HRV-synchrony to be maladaptive in the context of parental psychopathology or emotion regulation difficulties [42, 43]. Finally, recent research also points out that as children grow up, strong physiological coupling might be less adaptive and hinder the child’s abilities for self-regulation [41, 42].

As behavioral synchrony is a marker of healthy parent–child interaction not only in infancy but also adolescence [32, 34, 44], consistent co-occurrence of higher behavioral and HRV-synchrony may point to higher adaptiveness of the latter. However, the few studies examining these associations have shown that physiological and behavioral synchrony do not necessarily co-occur, and if they do, findings are inconsistent [37, 45]. An early study examining infants and mothers suggested that cardiac synchrony was higher when affect and vocal synchrony was also higher [46]. However, in another study during preadolescence, clinical dyads displayed the strongest HRV-synchrony combined with the lowest behavioral synchrony and resilient dyads displayed the lowest HRV-synchrony and the highest behavioral synchrony [41].

Dysregulated and unstable relationships with caregivers are common in adolescents with BPD [5, 47]. Yet, the underlying mechanisms, e.g. how behavioral synchrony, context and BPD pathology shape individual and HRV-synchrony during interaction are still unclear. We aimed at closing this gap by implementing a case–control design which included adolescents with at least subthreshold BPD (BPD-A) and their mothers (BPD-M) and a healthy comparison group of adolescents (HC-A) and their mothers (HC-M). We included resting periods as well as two interactional contexts of different valence (positive interaction vs. stress task). Hopefully, our results will contribute to a better understanding of the development of emotion dysregulation and interactional difficulties in BPD.

### Present study

With our paper, we aimed at adding knowledge on 1) individual resting state -, reactivity- and recovery- HRV in both adolescents and mothers 2) intrapersonal concordance of interactional quality and HRV, 3) HRV-synchrony and 4) relations between HRV-synchrony and behavioral synchrony. These associations were examined depending on clinical status and assessment context, i.e. resting, reactivity to positive and stressful interactions and recovery after stress.

Regarding individual HRV as research question (RQ) 1, we first hypothesized that BPD-A/BPD-M would display lower *resting state HRV* than HC-A/HC-M. We further assumed that *HRV-reactivity and -recovery* of adolescents and mothers would depend on the interplay between context and clinical status. We hypothesized that HC-A/HC-M would not show significant phasic HRV changes from *resting to positive interaction* but exhibit a decrease in HRV from *resting to stress task*, indicating attention and mobilization of resources [16]. As prior research has shown divergent results regarding HRV reactivity in adolescents with psychopathology, no specific hypotheses were made for BPD-A/BPD-M. For HRV-recovery we hypothesized that HC-A/HC-M would exhibit an increase in HRV (indicating disengagement from stressors and calmness) while BPD-A/BPD-M would not (based on the slower return-to-baseline findings). RQ2 zooms in on intrapersonal concordance, i.e. the link between interactional quality and HRV observed during mother-adolescent interaction. During *positive interaction*, we expected a positive link between individual interactional quality and HRV, suggesting that higher interactional quality supports higher HRV or the reverse. During *stress task*, we expected a negative link between individual interactional quality and HRV, suggesting that interactional quality supports effective regulation or effective regulation supports interactional quality. A moderating role of clinical status was examined exploratorily. Finally, we hypothesized that HRV-synchrony would be moderated by a) clinical status and context (RQ3) and b) behavioral synchrony and context (RQ4). In line with previous findings and theoretical considerations regarding interactional difficulties in BPD, we predicted that HC-M/HC-A show higher behavioral synchrony than BPD-M/BPD-A and that HC-M/HC-A and dyads higher in behavioral synchrony would not be HRV-synched during both interactions, while BPD-M/BPD-A and dyads lower in behavioral synchrony would be positively synched during both positive interaction and stress task.

## Methods

### Recruitment and participants

For a detailed description of recruitment procedure and the sample see Williams et al. [48]. In the present study, 38 adolescent patients (BPD-A; *mean*_*age*_ = 15.6, *sd*_*age*_ = 1.13) from a specialized outpatient clinic for self-harm and risk-taking behavior (AtR!Sk; [49]) and their mothers (BPD-M) were included. Clinical assessment of BPD-A was conducted by trained psychotherapists. The German version of the Structured Clinical Interview for DSM-5-Personality Disorders (SCID-5-PD; [50]) was used to assess borderline personality disorder criteria. BPD-A were included if they fulfilled the diagnostic criteria for subsyndromal BPD (≥ 3 BPD criteria) as adolescent subthreshold BPD is already associated with significant impairments in social and regulatory functioning [51]. In our sample, BPD-A met a minimum of three and a maximum of eight BPD criteria [*n*(3) = 8, *n*(4) = 11, *n*(5) = 9, *n*(6) = 7, *n*(7) = 2, *n*(8) = 2; *mean* = 4.74, *sd* = 1.41]. The healthy comparison sample consisted of 35 adolescents (HC-A; *mean*_*age*_ = 15.5, *sd*_*age*_ = 1.25) and their mothers (HC-M) with a similar distribution of adolescent sex, age, education and maternal education as the BPD dyads [48]. HC-A were excluded if they fulfilled criteria for any current or lifetime disorder according to the Mini International Neuropsychiatric Interview for Children and Adolescents [52]; HC-M were excluded if they had been in treatment at any mental health facility during the past two years.

All mothers were primary caregivers of the target adolescent. The sample was of European ancestry and well educated [48]. Most adolescents of both groups identified as female [(BPD-A: *n* = 32 (84.2%), HC-A: *n* = 28 (80.0%)]. Exclusion criteria for all participants were serious somatic illnesses, neurological disorders or dysfunctions of cardiac or hypothalamus–pituitary–adrenal systems.

### Procedure

The study was approved by the Institutional Review Board of the Medical Faculty, Heidelberg University.

Mothers and adolescents were invited to two laboratory visits. During the first visit, the clinical interviews were conducted. During the second visit, cardiac data was collected during a standardizes procedure including two interaction paradigms. Dyads gave written informed consent and were compensated 60 Euro for participation.

### Measures

#### Experimental paradigm

**Fig. 1:**
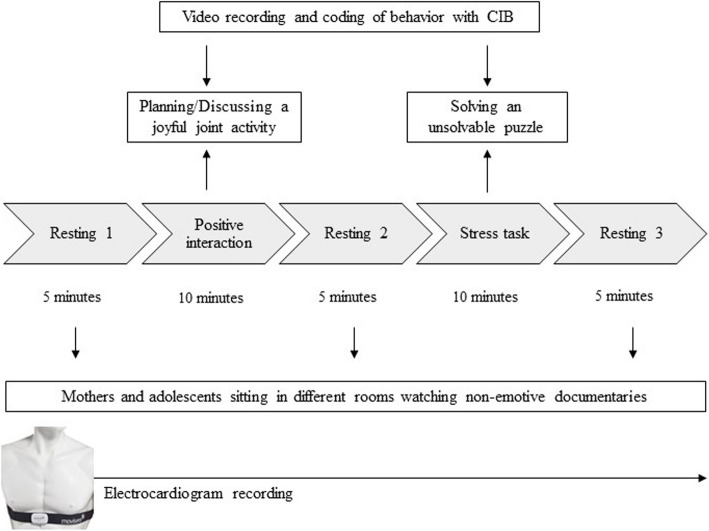
Description of the paradigm. *Note.* CIB = Coding interactive behavior manual

Participants completed five different segments in which continuous HRV was measured: resting 1, positive interaction, resting 2, stress task, and resting 3 (see Fig. 1). During the 5-min resting periods, mothers and adolescents sat quietly in different rooms and watched segments of non-emotive documentaries. The documentaries were purely factual, focusing on topics such as the types of clouds and their formation processes. They did not include any themes related to relationships, attachment, or emotions. During the 10-min long positive interaction, dyads were asked to plan and discuss a joyful joint activity. For the 10-min long stress task, adolescents were given a puzzle which was too difficult to solve in the provided timeframe. Additionally, they were informed that other participants their age had solved the task quickly and without any problems. Mothers were asked to support the adolescents, but not to solve the puzzle for them. For a more detailed description see also Williams et al. [48].

#### HRV measurement

Electrocardiogram recordings were collected using the ECGMove 3/4 sensors (movisens, Karlsruhe, Germany) which were attached to the sternum of participants using chest belts. Raw data were visually inspected in Kubios HRV premium (Version 3.1, Department of Applied Physics, University of Eastern Finland, Kuopio, Finland) and manually corrected. RHRV [53] was used to analyze the corrected interbeat intervall time series data. Root mean square of successive RR interval differences (rMSSD in ms) was calculated for each of the 35 one-minute segments. Due to HRV device malfunction, HRV data were missing from 3 mothers (BPD-M: *n* = 1, HC-M: *n* = 2) and 2 BPD-A. The HRV patterns of 2 BPD-M suggested supraventricular extrasystoles and were therefore excluded from the HRV analyses. Additionally, 2 adolescents (*n* = 1 per group) were excluded from HRV analyses due to abnormal HRV (e.g. < 50 heart beats per minute). Hence, 137 participants (93,84%; BPD-A: *n* = 35, BPD-M: *n* = 35, HC-A: *n* = 34, HC-M: *n* = 33) were included in individual HRV analyses. Due to moving artifacts, 199 (7.63%) of 2555 segments were missing for mothers and 195 (7.63%) were missing for adolescents.

#### Quality of interactional behavior and behavioral synchrony

Adolescent, maternal and dyadic behavior was observed during positive interaction and stress task and rated based on the Coding Interactive Behavior system (CIB; [54]). The CIB version for parent–child conversational paradigms covers 56 behavioral codes which receive ratings from 1 (low) to 5 (high). Maternal CIB was calculated by subtracting maternal intrusiveness from maternal sensitivity and structuring (Cronbach’s alpha = 0.81), adolescent CIB was calculated by subtracting adolescent withdrawal from engagement and compliance (Cronbach’s alpha = 0.92) and dyadic CIB/behavioral synchrony was calculated by subtracting constriction and tension from dyadic reciprocity, compatibility, and fluency (Cronbach’s alpha = 0.90). The combination of scales into a general score was guided by the following logic: scales have opposite directions; higher values on maternal sensitivity, for example, are considered ‘positive’ and higher values on maternal intrusiveness are considered ‘negative’. Subtracting opposite oriented scales is mathematically equivalent (up to a constant term) to inverting one scale before adding them together. Thus, higher scores on maternal, adolescent, or dyadic CIB/behavioral synchrony represent higher interactional quality. Interrater reliability was checked continuously during the rating process. Overall, rater agreement was 88%. Cohen’s Kappa was 0.77.

#### General adolescent psychopathology

The Strengths and Difficulties Questionnaire (SDQ; [55]) is a self-report questionnaire and was applied to assess emotional and behavioral problems in both adolescent groups (*α* = 0.89).

#### Covariates

Participants age, sex, BMI, smoking habits (yes/no) and physical activity in minutes per week (The International Physical Activities Questionnaire (IPAQ; [56]) were assessed through survey questions or interviews. Potentially cardioactive medication, such as certain antidepressants or beta-blockers, which are known to influence HRV, were documented.

### Analytic plan

All analyzes were done in R version 4.3.2.; significance level was set at α = 0.05. An rMSSD outlier analysis was performed (≥ 3SD from mean rMSSD) and all analyses were run with and without participants with outliers. To resolve skewness of raw rMSSD values, a natural log transformation was applied.

Given the nested structure of the data (individual analyses RQ1/2: rMSSD segment values nested within individuals, synchrony analyses RQ3/4: individuals nested within dyads) [57], RQs were investigated by implementing multilevel modeling (MLM; maximum likelihood estimation). Marginal means and contrasts were estimated using the emmeans package [58]. Unstandardized estimates are reported. Random-intercept (RQ1) and random-intercept and -slope (RQ2, 3,4) models were estimated using the lme4 package [59].

Depending on the RQ, the contrast between different assessment contexts were investigated. To examine reactivity, the contrast between the task (positive interaction or stress task) and the preceding resting period was of interest; for recovery, the contrast between the task and the following resting period was of interest. Tukey method was applied for *p*-value adjustment of the contrasts.

Specifically, for the analyses of HRV baseline differences, HRV-reactivity and -recovery (RQ1), random-intercept models with group, context and group x context interaction were calculated. Time of lab visit, age, sex, BMI, smoking, physical activities and medication were included as covariates (in adolescent model adolescent age, sex, BMI, etc. and in maternal model maternal age, BMI, etc., respectively). To test whether our results were specifically related to borderline symptomatology rather than to general psychopathology, we included the total score of the SDQ and the interaction term with context (SDQ*context) as predictors in the adolescent models.

For RQ2, two random-intercept, random-slope MLMs were set up: An adolescent model including a three-way interaction of adolescent CIB, clinical status and context predicting adolescent rMSSD (with time of lab visit, adolescent age, sex, BMI, smoking, physical activities and medication as covariates) and a maternal model including a three-way interaction of maternal CIB, clinical status and context predicting maternal rMSSD (with time of lab visit, maternal age, BMI, smoking, physical activities and medication as covariates). Each subject was allowed to have a different intercept and a random CIB slope.

For synchrony analyses (RQ3/4), multilevel state-trait modeling was applied, allowing for simultaneous estimation of between-dyad (BD) and within-dyad (WD) effects [37, 60]. Thus, associations of maternal and adolescent rMSSD are parsed on “trait” and “state” levels. Average (trait) rMSSD was calculated by grand-mean centering [60]. If mothers’ or adolescents’ average rMSSD across all one-minute segments was zero, this average was equivalent to the sample mean of mothers or adolescents, respectively. WD effects portray concurrent, in-the-moment associations [61] and capture whether mother and adolescent state rMSSD coordinate across the 35 one-minute measurements. State rMSSD was calculated by subtracting the individual’s average rMSSD from each of the 35 segment rMSSD values of that respective individual, thereby describing each individual’s fluctuations around their own average rMSSD. Thus, a state rMSSD value of zero represents the individual’s average rMSSD level [59], a positive state rMSSD value represents an increase in rMSSD with respect to an individual’s average rMSSD, and negative state rMSSD indexes a decrease. For a more detailed description of the method see e.g. Fuchs et al. [62].

For RQ3, two random-intercept, random-slope MLMs were run to establish presence or absence of HRV-synchrony across groups and contexts (Mother-to-Adolescent, MtA, including the covariates time of lab visit, adolescent age, sex, BMI, smoking, physical activities and medication; Adolescent-to-Mother, AtM, including the covariates time of lab visit, maternal age, BMI, smoking, physical activities and medication). Predictors were group, context, HRV state, HRV trait, and interaction terms were group x context, group x state, context x state, group x context x state. Observations were grouped by subject and each subject was allowed to have an individual state slope. To investigate RQ4, again two random-intercept, random-slope models were set up (AtM, MtA), controlling for covariates (following the logic of RQ3) and group. The predictors were context, HRV state, HRV trait, dyadic CIB, and interactions were context x state, context x CIB, state x CIB, state x context x CIB. The three-way interaction was evaluated at discrete values for dyadic CIB given by the average value, minus (lower) and plus (higher) one standard deviation. For RQ3/4, observations were grouped by subject and each subject was allowed to have an individual HRV state slope.

In order to examine to what extent non-findings are related to limited power when testing the hypotheses, sensitivity analyses for RQ1 (two-way interaction) and RQ3 (three-way interaction) were calculated to identify the smallest effect the study was powered to find (see Supplement for further details).

## Results

### Individual HRV (RQ1)

#### Resting state

For adolescents, there was a significant effect of clinical status (*t*(81.5) = 3.898, *p* < 0.001): BPD-A (*m* = 3.51, *CI* = 3.38 – 3.65) had lower resting rMSSD than HC-A (*m* = 3.88, *CI* = 3.75– 4.02). For mothers, there was no significant difference (*t*(76.7) = 0.98, *p* = 0.329) in resting rMSSD between BPD-M (*m* = 3.15, *CI* = 3.01 – 3.29) and HC-M (*m* = 3.25, *CI* = 3.10 – 3.39). Excluding outliers or including covariates did not change the results.

#### Reactivity

**Fig. 2:**
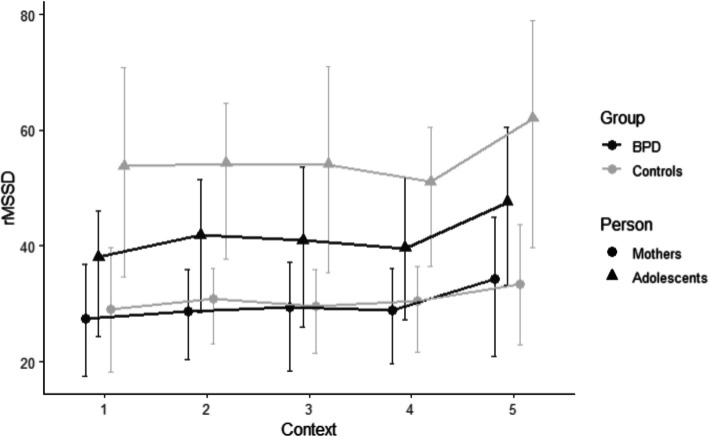
rMSSD reactivity/recovery in mothers and adolescents by measurement context and group. *Note.* Nontransformed rMSSD-values graphed. ** = significant changes, *p* <.01

For adolescents, a significant clinical status x context interaction [*F*(4,2250.25) = 2.886, *p* = 0.021] was found. Changes in rMSSD from *resting 1 to positive interaction* differed between groups: BPD-A showed a significant change of estimated marginal means (emm) from 3.55 (*SE* = 0.09) to 3.67 (*SE* = 0.09) (contrast: *β* = 0.12, *p* < 0.001) whilst in HC-A the rMSSD remained constant [change from 3.87 (*SE* = 0.11) to 3.89 (*SE* = 0.11); contrast: *β* = 0.02, *p* = 0.999]. Mothers showed a significant increase in rMSSD from *resting 1 to positive interaction* in both groups [BPD-M: changes from 3.10 (*SE* = 0.08) to 3.22 (*SE* = 0.08); contrast: *β* = 0.11, *p* < 0.001; HC-M: changes from from 3.17 (*SE* = 0.10) to 3.29 (*SE* = 0.10); contrast: *β* = 0.12, *p* < 0.001]. From *resting 2 to stress task*, no significant rMSSD changes or group differences could be observed (Table 1, Fig. 2). Sensitivity analysis suggests that the response to the stress task would need to be about the same size as the observed significant response to the positive interaction to be detected with sufficient power (see Supplement “Sensitivity Analysis RQ1” and Fig. S1). Since some observed responses in our sample meet the minimal resolvable effect size but the stress response is much smaller in magnitude, we do not interpret the absence of a stress response as a sample size issue.

**Table 1:** Clinical status and measurement context predicting individual rMSSD

	**Adolescent rMSSD**			**Mother rMSSD**		
*Predictors*	*Estimates*	*CI*	*p*	*Estimates*	*CI*	*p*
(Intercept)	3.537	3.277 – 3.796	**<0.001**	3.221	3.054 – 3.387	**<0.001**
Context2	0.121	0.075 – 0.168	**<0.001**	0.115	0.071 – 0.158	**<0.001**
Context3	0.092	0.038 – 0.145	**0.001**	0.098	0.047 – 0.148	**<0.001**
Context4	0.073	0.027 – 0.119	**0.002**	0.114	0.071 – 0.157	**<0.001**
Context5	0.256	0.203 – 0.310	**<0.001**	0.233	0.183 – 0.284	**<0.001**
Group (BPD/HC)	0.321	0.112 – 0.530	**0.003**	0.066	−0.137 – 0.269	0.525
Time of day	−0.024	−0.062 – 0.014	0.212	−0.031	−0.071 – 0.009	0.131
Age	−0.066	−0.145 – 0.014	0.106	−0.010	−0.027 – 0.007	0.249
Medication yes/no	0.104	−0.180 – 0.389	0.472	−0.055	−0.309 – 0.198	0.669
Physical activity	0.000	−0.000 – 0.000	0.869	−0.000	−0.000 – 0.000	0.380
BMI	−0.008	−0.036 – 0.019	0.564	−0.000	−0.021 – 0.021	0.985
Sex	0.042	−0.183 – 0.266	0.717			
Smoking yes/no	−0.117	−0.346 – 0.112	0.316	−0.174	−0.394 – 0.046	0.120
Context2:HC	−0.102	−0.170 – −0.033	**0.004**	0.006	−0.057 – 0.070	0.844
Context3:HC	−0.084	−0.163 – −0.006	**0.035**	−0.030	−0.104 – 0.043	0.423
Context4:HC	−0.105	−0.174 – −0.037	**0.003**	−0.011	−0.074 – 0.053	0.744
Context5:HC	−0.114	−0.192 – −0.035	**0.005**	−0.052	−0.126 – 0.022	0.167
**Random Effects**						
σ^2^	0.06			0.06		
τ_00_	0.12 _id_			0.14 _id_		
ICC	0.66			0.71		
N	69 _id_			68 _id_		
Observations	2318			2279		
Marginal R^2^ / Conditional R^2^	0.180 / 0.718			0.100 / 0.740		

Reference category: Context1= Resting 1. Context2 = Positive Interaction, Context3 = Resting 2, Context4 = Stress Task, Context5 = Resting 3. BPD = clinical group; HC = healthy controls

#### Recovery

Adolescents (*β* = 0.18, *SE* = 0.02, *p* < 0.001) and mothers (*β* = 0.10, *SE* = 0.02, *p* < 0.001) showed a significant increase in rMSSD from *stress task to resting 3*, irrespective of group [BPD-A: 3.62 (*SE* = 0.09) to 3.80 (*SE* = 0.09); HC-A: 3.84 (*SE* = 0.11) to 4.01 (*SE* = 0.11); BPD-M: 3.22 (*SE* = 0.08) to 3.34 (*SE* = 0.08); HC-M: 3.27 (*SE* = 0.10) to 3.35 (*SE* = 0.10)].

Analyses controlling for SDQ and SDQ*context interaction revealed that for resting state and recovery HRV, results remained robust. For HRV reactivity, results also stayed the same from resting 1 to positive interaction. From resting 2 to the stress task, however, significant differences were found within both groups dependent on the SDQ value: higher SDQ scores were associated with higher HRV differences between contexts (*b* = 0.01, *p* = 0.002). Specifically, HRV differences mainly represented lower HRV scores during stress. Plots for these additional calculations can be found in the supplement (Figs. S2-S4).

### Intrapersonal concordance of HRV and behavior (RQ2)

In the adolescent model, plotted regression lines (see supplement Fig. S5) suggested a negative CIB-rMSSD association for BPD-A and a positive CIB-rMSSD association for HC-A during the positive interaction (whilst both groups showed a negative regression line during stress). However, when outliers were excluded, the adolescent three-way interaction did not reach significance (*F*(3, 1415) = 2.60, *p* = 0.051). In the maternal model, a significant three-way interaction was found (*F*(3, 842) = 3.37, *p* = 0.018; see supplement Table S1 for results without outliers). For HC-M, plotting (Fig. S5) suggested negative CIB-rMSSD associations during stress task and positive CIB-rMSSD associations during positive interaction. However, only the positive association between CIB and rMSSD during positive interaction turned out to be significant (*β* = 0.13, *p* = 0.048).

### HRV-synchrony during caregiver-child interactions (RQ3)

**Fig. 3:**
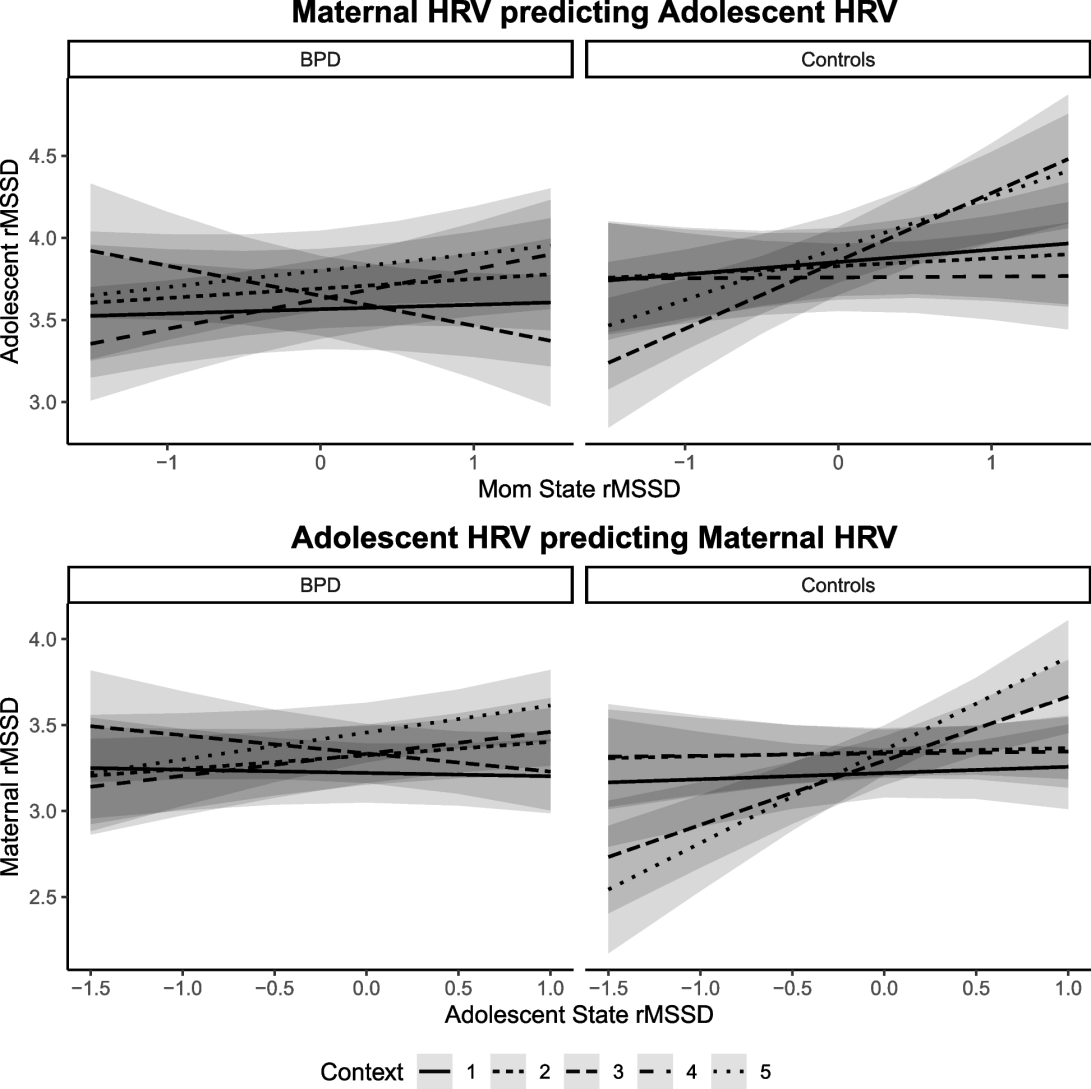
Clinical status and measurement context shape HRV-synchrony. *Note.* Context 1, 3, 5 = Resting: Context 2 = Positive interaction, Context 4 = Stress task. Significant synchrony only in BPD dyads during stress task (maternal HRV to adolescent HRV model, trend level in adolescent HRV to maternal HRV model). In control dyads, significant state HRV associations during rest 3 and 5 in both models

In MtA and AtM models, significant three-way interactions of state, clinical status and context were found (MtA: *F*(4, 1966) = 5.61, *p* < 0.001; AtM: *F*(4, 1963) = 5.58, *p* < 0.001; Table 2, Fig. 3). In the MtA-model, significant positive associations between state rMSSD in HC-A/HC-M (meaning positive synchrony) were found during resting 2 (*β* = 0.41, 9*5% CI* [0.19, 0.64]) and 3 (*β* = 0.31, 9*5% CI* [0.11, 0.52]), while in BPD-A/BPD-M significant positive synchrony was observed during the stress task (*β* = 0.18, 9*5% CI* [0.02, 0.34]). Thus, when mothers increased or decreased their rMSSD at any given moment during resting 2 or 3 (CG) or during stress (BPD), adolescents also increased or decreased their rMSSD with respect to their average rMSSD. Sensitivity analysis shows that, in the positive interaction context, the minimal synchrony required to be detected with sufficient power is about 1.5 times the observed synchrony during the stress task (see Supplement “Sensitivity analysis RQ3” and Fig. S6). Since the observed synchrony effects are of the same order as the minimal resolvable effect size but are 5.8 times smaller, we do not attribute the absence of synchrony during the positive interaction to a sample size issue. In the AtM-model, significant positive state rMSSD synchrony was found in HC-A/HC-M during resting 2 (*β* = 0.37, 9*5% CI* [0.19, 0.56]) and 3 (*β* = 0.54, 9*5% CI* [0.34, 0.74]), however, there was only trend-level significance of synchrony in BPD-A/BPD-M during stress task (*β* = 0.13, 9*5% CI* [0.00, 0.26], *p* = 0.06). Again, there was no association between adolescent and maternal average rMSSD in either model.

**Table 2:** Clinical status and measurement context shape HRV-synchrony

	**Mother rMSSD -> Adolescent rMSSD**			**Adolescent rMSSD -> Mother rMSSD**		
*Predictors*	*Estimates*	*CI*	*p*	*Estimates*	*CI*	*p*
(Intercept)	3.566	3.323 – 3.808	**<0.001**	3.224	3.051 – 3.396	**<0.001**
Time of day	−0.020	−0.057 – 0.016	0.271	−0.014	−0.053 – 0.025	0.480
Age	−0.083	−0.162 – −0.003	**0.042**	−0.013	−0.030 – 0.004	0.130
Medication yes/no	0.047	−0.224 – 0.318	0.735	0.024	−0.221 – 0.269	0.847
Physical activity	0.000	−0.000 – 0.000	0.716	−0.000	−0.000 – 0.000	0.745
BMI	−0.002	−0.028 – 0.024	0.882	−0.006	−0.026 – 0.014	0.540
Smoking yes/no	−0.158	−0.377 – 0.060	0.156	−0.163	−0.385 – 0.059	0.150
Sex	0.067	−0.141 – 0.276	0.526			
Average rMSSD	0.123	−0.095 – 0.340	0.270	0.243	−0.017 – 0.502	0.067
State rMSSD	0.027	−0.170 – 0.224	0.786	−0.019	−0.166 – 0.128	0.798
Group (BPD/HC)	0.287	0.082 – 0.493	**0.006**	−0.000	−0.216 – 0.215	0.997
Context2	0.126	0.074 – 0.178	**<0.001**	0.102	0.055 – 0.150	**<0.001**
Context3	0.082	0.024 – 0.140	**0.006**	0.114	0.062 – 0.166	**<0.001**
Context4	0.061	0.010 – 0.113	**0.020**	0.111	0.065 – 0.158	**<0.001**
Context5	0.235	0.172 – 0.298	**<0.001**	0.235	0.178 – 0.293	**<0.001**
State:HC	0.048	−0.233 – 0.329	0.738	0.055	−0.208 – 0.319	0.680
State:Context2	0.031	−0.194 – 0.255	0.789	0.098	−0.071 – 0.267	0.256
State:Context3	−0.210	−0.467 – 0.047	0.110	−0.087	−0.275 – 0.102	0.368
State:Context4	0.154	−0.068 – 0.377	0.174	0.147	−0.018 – 0.312	0.081
State:Context5	0.073	−0.153 – 0.300	0.525	0.176	−0.019 – 0.371	0.076
HC:Context2	−0.151	−0.229 – −0.072	**<0.001**	0.019	−0.050 – 0.088	0.582
HC:Context3	−0.075	−0.162 – 0.011	0.089	−0.042	−0.119 – 0.035	0.286
HC:Context4	−0.155	−0.233 – −0.077	**<0.001**	0.001	−0.068 – 0.071	0.967
HC:Context5	−0.151	−0.244 – −0.058	**0.001**	−0.102	−0.186 – −0.018	**0.017**
State:HC:Context2	−0.058	−0.381 – 0.264	0.723	−0.111	−0.413 – 0.192	0.473
State:HC:Context3	0.549	0.172 – 0.926	**0.004**	0.424	0.106 – 0.742	**0.009**
State:HC:Context4	−0.224	−0.551 – 0.103	0.179	−0.171	−0.484 – 0.141	0.282
State:HC:Context5	0.166	−0.181 – 0.513	0.350	0.328	−0.003 – 0.660	0.052
**Random Effects**						
σ^2^	0.06			0.05		
τ_00_	0.10 _id_			0.14 _id_		
τ_11_	0.06 _id.rMSSDlb_state_			0.04 _id.rMSSDlj_state_		
ρ_01_	−0.16 _id_			−0.42 _id_		
ICC	0.64			0.73		
N	64 _id_			64 _id_		
Observations	2049			2049		
Marginal R^2^ / Conditional R^2^	0.227 / 0.720			0.107 / 0.758		

Reference category: Context1 = Resting 1. Context2 = Positive Interaction, Context3 = Resting 2, Context4 = Stress Task, Context5 = Resting 3. 64 dyads, 2049 observations. BPD = clinical group; HC = healthy controls

### Relations between interpersonal HRV- and behavioral synchrony (RQ4)

In both models, significant three-way interactions of state rMSSD, dyadic CIB as an index of behavioral synchrony and context were found (MtA: *F*(3, 1561) = 5.36, *p* = 0.001; AtM: *F*(3, 1565) = 4.78, *p* = 0.003). When dyadic CIB was observed to be *lower*, significant positive HRV-synchrony was found in both models during stress (MtA: *β* = 0.16, *p* = 0.024; AtM: *β* = 0.15, *p* = 0.020). In the MtA model, significant positive state rMSSD associations were also found during resting 2 and 3 when dyadic CIB was observed to be *average* (3: *β* = 0.22, *p* < 0.001) and *higher* (2: *β* = 0.43, *p* < 0.001; 3: *β* = 0.33, *p* < 0.001). In the AtM model, significant positive state rMSSD synchrony was found during resting 2 when dyadic CIB was *higher* (*β* = 0.25, *p* = 0.010), and during resting 3 across all levels of behavioral synchrony (*higher*: *β* = 0.46, *p* < 0.001: *average*: *β* = 0.31, *p* < 0.001, *lower: β* = 0.16, *p* = 0.040). There was no association between adolescent and maternal average rMSSD in either model. See supplement Table S2 and Fig. S7 for visualization.

The finding of positive HRV-synchrony during rest in CG-dyads and dyads average to higher in behavioral synchrony was surprising, given that synchrony is considered to be tied to a social process which should first and foremost be present when partners are concurrently interacting and not when they are sitting apart resting. To rule out the possibility that these synchrony findings emerged only because adolescents and mothers showed similar trajectories during rest independent from any dyad-specific social processes, we exploratively shuffled mothers and adolescents within clinical groups and randomly assigned mother-adolescent pairs. Again, two random intercept, random slope multilevel models including the covariates start time, age, sex adolescent, medication, physical activity, BMI, and smoking were run. Clinical status and context were added as moderators of HRV-Synchrony to both AtM and MtA-models. Contrary to findings from the original dataset, three-way interactions of state, clinical status and context were not significant (MtA: *F*(4, 1821) = 0.47, *p* = 0.758; AtM: *F*(4, 1947) = 0.73, *p* = 0.571; Table S3), suggesting that the reported effect was not significant in randomly assigned mother-adolescent pairs.

## Discussion

With our study, we aimed at examining physiological regulation and interactional behavior during rest and positive and stressful contexts in order to shed light on real-time individual and dyadic regulation in adolescents with BPD traits and their mothers. We examined 1) individual HRV -resting state, -reactivity and -recovery 2) intrapersonal concordance of interactional quality and HRV, 3) HRV-synchrony and 4) relations between HRV-synchrony and behavioral synchrony.

### Individual HRV (RQ1)

As hypothesized and in line with previous research, BPD-A showed a decreased *resting HRV* in comparison to HC-A. This result was not influenced by general psychopathology which is surprising given the fact that lower resting state HRV was found in a variety of mental disorders (e.g. [63]). For mothers, no group differences were found. Regarding *HRV-reactivity*, we found significant HRV-changes over time, suggesting that the measurement of HRV was sensitive to environmental or contextual changes. BPD-A and mothers of both groups showed a significant increase in HRV from *resting to positive interaction*, suggesting a state of relaxation and calmness during social interaction*.* For the BPD group, there is also an alternative explanation: BPD-A/BPD-M may not have experienced their interaction as positive due to their history of conflict-ridden exchanges often observed in adolescents with BPD and their parents [5]. Therefore, the HRV-increase may reflect heightened self-regulatory efforts or/and the use of emotion suppression as an emotion regulation strategy, two mechanisms that were previously associated with BPD and HRV-augmentation [27, 64, 65]. The HRV-increase was not significant for the HC-A, a finding that would be in line with evidence from prior studies based mostly on community samples suggesting that neutral or positive social interactions may not induce changes in phasic HRV [16]. However, the HRV-increase observed in BPD-A and BPD-M/HC-M may also simply be a consequence of lower resting HRV at the beginning of the visit and indicate a process of recuperation during dyadic interaction. Despite of having spent about 30 min at the laboratory prior to the first resting assessment, mothers and BPD-A may have still been influenced by a feeling of nervousness, whereas HC-A may have been rather undeterred by the assessment context. Effects were independent of general adolescent psychopathology and therefore appear to be specific to BPD symptomatology.

HRV-reactivity to the stress task did not reach significance in our sample. One obvious explanation may be that the task simply was not stressful enough, however, subjective reports from mothers and adolescents of positive and negative affect suggest otherwise: both reported significantly more negative affect [mothers: *t*(72) = −5.731, *p* < 0.001; adolescents: *t*(72) = −6.706, *p* < 0.001] and less positive affect (mothers: *t*(72) = 2.66, *p* < 0.01; adolescents: *t*(72) = 3.15, *p* < 0.01) immediately after the stress task compared to their reports immediately following the positive interaction. We also could find differences in behavior between the positive interaction and the stress task [48]. The lack of reactivity may be explained by different mechanisms. Prior studies suggest that while a decrease in HRV in response to stress seems to be normative in low-risk children, children at risk for psychopathology or with mental disorders may suffer from a lack of autonomic flexibility [66] which could have effected stress responding in BPD-A. These findings are consistent with prior research that could not confirm the hypothesis of physiological hyperreactivity in individuals with BPD [23, 24]. In HC-A, supportive presence of mothers and/or higher behavioral skills [48] may have potentially dampened HRV stress reactivity. However, we did find differences in stress reactivity based on general psychopathology scores. This suggests that stress reactivity may be influenced more by general emotional and behavioral problems than by BPD-specific symptoms. Future research should include individuals with other mental disorders to further investigate this aspect. Regarding the mothers of our sample, a lack of HRV-reactivity could be explained by the task design, as the stress task mainly targeted adolescents and mothers were only asked to support their youth. Although maternal self-reports and behavioral observations suggest that stress was perceived, this did not manifest as a physiological stress response. However, as there is first evidence of significant associations between maternal HRV, maternal interactional quality and child and adolescent outcomes [26–28], more research is needed to disentangle processes of HRV-reactivity and their consequences for maternal behavior and adolescent outcome. Lastly, our hypothesis of group differences in *HRV-recovery* was not confirmed, as both BPD-A/-M and HC-A/-M demonstrated a significant HRV-increase from stress to resting [16] (which could also have been influenced by the anticipated near end of the experimental paradigm).

### Intrapersonal concordance of HRV and behavior (RQ2)

HC-A/HC-M displayed the expected positive association of rMSSD and behavioral quality during positive interaction (indicating a regulated, relaxed state of mind) and the negative association of rMSSD and behavior quality during stress (indicating engagement with stress, adaptive coping and efficient self-regulation). Although only the HC-M result during positive interaction became significant, these (trend) findings are in line with previous considerations [9, 10]. Behavior-rMSSD concordances in BPD-A/M were not significant. However, plotting suggested that a) BPD-A showed the same pattern during positive interaction as during stress (which could indicate that even the positive caregiver-adolescent interaction requires active self-regulation) and b) rMSSD and behavior of BPD-M seem to be completely independent from each other, which is an interesting result considering the role of an invalidating environment in the development of BPD [2]. During early childhood, children turn towards their parents to understand the nature of a situation (e.g. if the situation is stressful or potentially harmful) and how to appropriately regulate their upcoming emotions in this particular situation. However, perceiving diverging physiological and behavioral information from caregivers, which serve as a source of co-regulation, can disrupt the child’s ability to develop effective self-regulatory skills, potentially leading to difficulties in self-regulation later in life.

Our findings on the individual concordance between behavior and HRV should be interpreted with caution. While plots suggest certain trends, statistical analyses in our study confirmed only one of these assumptions. More research is needed to illuminate links between behavior and physiology during parent–child interaction especially in adolescent clinical samples.

### HRV- and behavioral synchrony between adolescents and their parents (RQ3/4)

In line with our hypothesis, we found that mother-to-adolescent (MtA) and adolescent-to-mother (AtM) HRV-synchrony were significantly moderated by the interplay of clinical status or behavioral synchrony, respectively, and context. Overall, CG-dyads or dyads higher in behavioral synchrony were positively synchronized during rest and BPD-dyads or dyads lower in behavioral synchrony during the stress task.

Behavioral synchrony has been hypothesized to be a sign for healthy parent-adolescent interactions [32, 34, 44]. Motsan et al. [41] suggested that during adolescence, tight autonomic coupling might have to be replaced by more “loosely” coordinated behavioral exchanges, reflecting the growing importance of developing autonomy in this phase. In a healthy, age-appropriate development, the adolescent might have already developed the ability to self-regulate and self-confidence in own abilities to solve potentially stressful situations resulting in greater independence from parental physiology. The fact that we also did not find HRV-synchrony during interactions in dyads higher in behavioral synchrony would support this interpretation: if dyads are able to rely on their behavioral abilities, no physiological co-regulation might be necessary. A closer look at our behavioral data during interactions [48] indicates an increase in reciprocal behavior during stress in our healthy dyads, which would also point towards co-regulating on a behavioral rather than a physiological level. HRV-synchrony in CG-dyads while resting in separate rooms right after having completed the interactional tasks appeared to be dyad-specific and the effect vanished when adolescents were randomly assigned to unfamiliar mothers: similar socio-emotional post-processing of the collective prior experience may allow for HRV-synchrony to emerge [39].

As for the BPD-dyads, a history of tumultuous and chaotic interactions might have decreased trust into their respective partner’s ability to co-regulate on a behavioral level. Mothers might have experienced frequent anger outbursts and unpredictability in their adolescents which might cause hypervigilance and/or withdrawal on a behavioral level. Adolescents, on the other hand, might not have developed the abilities to emotionally or behaviorally self-regulate (a common symptom in BPD), which could throw them into a state of hypervigilance, despair and inability to access cognitive processes that would be helpful in solving the stressful situation (which could e.g. also involve asking the parent for help). BPD-dyads therefore might “fall back” into a more basic co-regulating system, the physiological co-regulation. Again, our behavioral data supports this line of thought by indicating a decrease in dyadic reciprocity during stress in BPD-dyads [48]. These considerations are also supported by the fact that we found the same HRV-synchrony pattern in dyads low or medium in behavioral synchrony.

Future research has to determine if positive HRV-synchrony in this context is adaptive (maybe physiological attunement did in fact calm the adolescent) or maladaptive (leading to a physiological stress escalation). This research question could be explored by, for example, investigating how physiological synchrony and subjective reports of stress are related to each other. Also, longitudinal data should focus on the interplay of physiological and behavioral synchrony, how it develops over time and how disruptions in the normal process could add to clinical symptoms. This is especially important in disorders like BPD, that was repeatedly described as an emotion regulation disorder at its core (and also as a disorder of failed co-regulation; 6), as it might open potential windows for early interventions, preventing the development of emotion regulation disorders (e.g., by focusing on dyadic behavior in parent–child dyads).

### Limitations

While our study has several strengths such as the implementation of a case–control design, observed behavioral and physiological measures and a unique sample of adolescents with BPD traits and their mothers, there are some limitations to discuss (see also [48]). First and as mentioned above, although subjective reports suggested that our stress task did induce negative emotions and stress, these may not have sufficed to trigger responses of the autonomous nervous system. Also, averaging rMSSD-values over one-minute segments and using a global behavior coding system behavior may have masked more fine-grained patterns of behavior-physiology- or physiology-physiology-associations. It will be interesting to see whether present results can be replicated in datasets including micro-coded behavior and second-by-second HRV. Despite all its advantages, concurrent state-trait MLM does not allow for examination of mother-to-adolescent or adolescent-to-mother directionality. Furthermore, other operationalizations of synchrony, i.e. the definition of linkage between mother and adolescent HRV, can lead to different results. Since parent-adolescent interactional quality is often reduced in clinical dyads, larger and more diverse samples are needed to inform on these effects. Lastly, we did not assess maternal BPD symptomatology and therefore cannot exclude potential influences on maternal behavior and/or physiology.

## Conclusions

Our study is the first to suggest alterations in HRV-reactivity, behavior-HRV concordance and HRV-synchrony in adolescents with BPD traits and their mothers. While we were able to confirm some prior research findings (e.g., lower resting HRV in adolescents with BPD, no HRV change in HC-A from resting to positive interaction, dampened HRV response to stress in BPD-A), many questions remain and more data is needed to replicate current results. As our study cannot definitively determine whether the results are specific to BPD or applicable to various psychiatric disorders (or transdiagnostic symptoms like emotion dysregulation), future studies should include multiple diagnostic groups alongside a healthy control group. Future research should investigate HRV reactivity to stress with established stress tasks (for example the Trier Social Stress Test, TSST; [67]) to ensure validity of results and eliminate concerns about whether stress was effectively induced. Especially our findings regarding the individual concordance of behavior and HRV warrant further exploration, as most of our results did not reach statistical significance. Such studies could provide valuable insights into the development of emotion regulation difficulties during childhood and adolescence.

Moreover, our findings of physiological synchrony during stress—in BPD dyads and dyads with low behavioral synchrony, but not in healthy dyads and dyads with high behavioral synchrony—highlight the importance of exploring biobehavioral synchrony as a theoretical framework to advance our understanding of caregiver-child co-regulation processes in the context of a healthy versus pathological developmental pathways.

In order to establish whether the observed associations and their adaptiveness constitute a mechanism of BPD symptom development, further research should also investigate if and how existing BPD specific treatment options influence behavioral and physiological parent-adolescent synchrony. Of specific interest could be therapies focusing on the enhancement of interactional and emotion regulation skills (e.g., Dialectic Behavioral Therapy for Adolescents, DBT-A; [68]). Furthermore, the current study aligns with existing guidelines (such as those of the German Association of the Scientific Medical Societies AWMF in Germany, [69]; or the National Institute for Health and Care Excellence NICE in the UK, [70]), as well as treatments like DBT-A [68] that address adolescent BPD pathology not in isolation but as part of a broader social system, recognizing the importance of daily dyadic interactions in which adolescents engage. In the long run, a thorough understanding of individual and dyadic bio-behavioral regulation in the context of parent-adolescent interactions may support clinicians in working with BPD-adolescents and could help to conceptualize effective treatment approaches which specifically target interpersonal regulation.

## Supplementary Information

Supplementary Material 1. 

## Data Availability

The dataset used during the current study is available from the corresponding author on reasonable request.

## Abbreviations

BPD: Borderline personality disorder
ER: Emotion regulation
HRV: Heart rate variability
BPD-A: Clinical group of adolescents
BPD_M: Clinical group of mothers
HC-A: Healthy control group of adolescents
HC-M: Healthy control group of mothers
PTSD: Post traumatic stress disorder
RQ: Research question
SD: Standard deviation
SCID-5-PD: Structured clinical interview for DSM-5-Personality Disorders
CIB: Coding interactive behavior
rMSSD: Root mean square of successive RR interval differences
BMI: Body mass index
MLM: Multilevel modeling
WD: Within-dyad
MtA: Mother to adolescent
AtM: Adolescent to mother
