# Supplementary material for: Individual patterns and synchrony of heart rate variability in adolescent patients with borderline personality psychopathology and their mothers: a case–control study
Source: Borderline Personal Disord Emot Dysregul. 2025 Apr 9;12:12. doi: 10.1186/s40479-025-00289-0 (PMC11980296; doi:10.1186/s40479-025-00289-0)
Supplement: Supplementary file 1 — Supplementary Material 1. [file 40479_2025_289_MOESM1_ESM.docx]

**Supplement Material**

**Sensitivity analysis RQ1**

This study had a given sample size, including 35 control dyads and 38 clinical dyads. As we do not find a stress response in HC-A, we did a sensitivity analysis with a bootstrapped control sample. In order to calculate the smallest response the study was powered to find, we artificially tuned the stress response in RQ1 for adolescents, i.e. the two-way interaction Group x Context, by adding an outcome contribution for HC-A in the stress context. The effect sizes were thus not standardized but are reported in units of the outcome (logarithmized rMSSD). They are tuned in the direction of the observed effect.

We used 1000 bootstrapped samples to approximate the power for each effect size. An additional contribution to the log-transformed adolescents HRV was subtracted in each sample in the stress context for controls. Afterwards, the model was recalculated. Power corresponds to the proportion of significant stress responses (contrast between stress context and resting 2) within the 1000 analyses.

For a power of 0.8 at a significance level of α=.05, analyses revealed an effect size of about 0.125 for the stress response in HC (Figure S1). This suggests that the stress response needs to be about the same size of the observed response to the positive interaction in the clinical group to be resolved with enough power. Since some observed responses in our sample meet the minimal resolvable effect size but measure a 3.2 times smaller effect, we do not attribute the absence of a stress response to a sample size issue.

**Sensitivity analysis RQ3**

This study had a given sample size, including 35 control dyads and 38 clinical dyads. As we do not find synchrony in the positive interaction task for controls, we did a sensitivity analysis with a bootstrapped control sample. In order to calculate the smallest response the study was powered to find, we artificially tuned the synchrony in RQ 3 in the MtA model, i.e. the three-way interaction group x context x mothers state rMSSD, by adding an outcome contribution for the controls in the positive interaction context proportional to mothers state rMSSD. The effect sizes were thus not standardized but are reported in units of one. They are tuned in the direction of the observed effect.

We used 1000 bootstrapped samples to approximate the power for each effect size. An additional contribution to the log-transformed adolescents HRV was added in each sample in the stress context for controls. Afterwards, the model was recalculated. Power corresponds to the proportion of significant synchrony coefficients (dependency on mothers’ state rMSSD for controls in the positive interaction) within the 1000 analyses.

For a power of 0.8 at a significance level of α=.05, analyses revealed an effect size of about 0.275 (Figure S2). This suggests that the synchrony needs to be about 1.5 times the synchrony observed during stress task in the clinical group to be resolved with enough power. As we do see synchrony effects of the same order as the minimal resolvable effect size, but measure a 5.8 times smaller effect, we don’t consider the missing synchrony during the positive interaction a sample size problem.

**Tables**

**Table S1**

*Observed behavior during interaction, clinical status and context predicting individual HRV*

|  | **Adolescent RMSSD** | | | **Mother RMSSD** | | |
| --- | --- | --- | --- | --- | --- | --- |
| *Predictors* | *Estimates* | *Conf. Int. (95%)* | *P-Value* | *Estimates* | *Conf. Int. (95%)* | *P-Value* |
| Intercept | 3.923 | 3.450 – 4.396 | **<0.001** | 3.298 | 3.066 – 3.529 | **<0.001** |
| Time of day | -0.028 | -0.071 – 0.014 | 0.187 | -0.030 | -0.069 – 0.010 | 0.137 |
| Context3 | -0.022 | -0.160 – 0.116 | 0.755 | -0.090 | -0.240 – 0.060 | 0.240 |
| Context4 | -0.166 | -0.301 – -0.032 | **0.015** | -0.004 | -0.177 – 0.169 | 0.962 |
| Context5 | -0.017 | -0.1734– 0.140 | 0.833 | 0.096 | -0.111 – 0.301 | 0.357 |
| Group (BPD/HC) | -0.165 | -0.838 – 0.507 | 0.623 | -0.211 | -0.620 – 0.199 | 0.309 |
| Behavior | -0.136 | -0.303 – 0.030 | 0.106 | -0.002 | -0.072 – 0.067 | 0.944 |
| Age | -0.060 | -0.152 – 0.031 | 0.193 | -0.011 | -0.027 – 0.006 | 0.190 |
| Medication yes/no | 0.070 | -0.266 – 0.407 | 0.677 | 0.002 | 0.243 – 0.246 | 0.988 |
| Physical activity | 0.000 | -0.000 – 0.000 | 0.444 | -0.000 | -0.000 – 0.000 | 0.167 |
| BMI | -0.004 | -0.035 – 0.026 | 0.779 | -0.001 | -0.021 – 0.020 | 0.940 |
| Smoking yes/no | -0.103 | -0.362 – 0.156 | 0.429 | -0.116 | -0.330 – 0.098 | 0.284 |
| Sex | 0.038 | -0.215 – 0.291 | 0.766 | - | - | - |
| Context3:HC | 0.342 | 0.028 – 0.657 | **0.033** | 0.004 | -0.331 – 0.340 | 0.980 |
| Context4:HC | 0.432 | 0.030 – 0.835 | **0.035** | 0.489 | 0.161 – 0.818 | **0.004** |
| Context5:HC | 0.500 | 0.015 – 0.984 | **0.043** | 0.084 | -0.322 – 0.489 | 0.685 |
| Context3:Behavior | -0.004 | -0.069 – 0.062 | 0.916 | 0.036 | -0.035 – 0.108 | 0.315 |
| Context4:Behavior | 0.048 | -0.018 – 0.114 | 0.154 | 0.001 | -0.083 – 0.085 | 0.979 |
| Context5:Behavior | 0.059 | -0.018 – 0.136 | 0.134 | 0.002 | -0.099– 0.103 | 0.962 |
| HC:Behavior | 0.193 | -0.082– 0.468 | 0.165 | 0.135 | -0.013 – 0.283 | 0.073 |
| Context3:HC:Behavior | -0.135 | -0.271 – 0.001 | 0.052 | -0.025 | -0.169 – 0.120 | 0.740 |
| Context4:HC:Behavior | -0.192 | -0.366 – -0.017 | **0.031** | -0.230 | -0.378 – -0.081 | **0.002** |
| Context5:HC:Behavior | -0.225 | -0.436– -0.0124 | **0.037** | -0.057 | -0.241 – 0.127 | 0.542 |
| **Random Effects** | | | | | | |
| Intercept Variance | 0.84 _id_ | | | 0.22 _id_ | | |
| Behavior Variance | 0.14 _id/behavior_ | | | 0.00 _id/behavior_ | | |
| ICC | 0.77 | | | 0.72 | | |
| N | 69 _id_ | | | 68 _id_ | | |
| Observations | 1944 | | | 1899 | | |
| Marginal / Conditional R^2^ | 0.145 / 0.805 | | | 0.094 / 0.750 | | |

*Note.* Reference category: Context2=Positive Interaction, Context3 =Resting 2, Context4=Stress Task, Context5=Resting 3. Behavior=Total CIB score: Maternal positive behavior predicting maternal vmHRV, Adolescent positive behavior predicting adolescent vmHRV. BPD = clinical group; HC = healthy controls.

**Table S2**

*Behavioral synchrony and measurement context shape vmHRV synchrony*

|  | **Mother RMSSD -> Adolescent RMSSD** | | | **Adolescent RMSSD -> Mother RMSSD** | | |
| --- | --- | --- | --- | --- | --- | --- |
| *Predictors* | *Estimates* | *Conf. Int. (95%)* | *P-Value* | *Estimates* | *Conf. Int. (95%)* | *P-Value* |
| Intercept | 3.739 | 3.481 – 3.997 | **<0.001** | 3.304 | 3.130 – 3.477 | **<0.001** |
| Average rMSSD | 0.094 | -0.137 – 0.325 | 0.419 | 0.263 | -0.000 – 0.526 | 0.050 |
| Group(BPD/HC) | 0.187 | -0.023 – 0.397 | 0.080 | 0.001 | -0.204 – 0.205 | 0.994 |
| State rMSSD | 0.063 | -0.152 – 0.278 | 0.566 | 0.089 | -0.071 – 0.249 | 0.274 |
| Context3 | -0.031 | -0.091 – 0.028 | 0.298 | 0.035 | -0.021 – 0.092 | 0.220 |
| Context4 | -0.071 | -0.116 – -0.025 | **0.003** | 0.021 | -0.022 – 0.064 | 0.347 |
| Context5 | 0.104 | 0.047 – 0.161 | **<0.001** | 0.130 | 0.075 – 0.185 | **<0.001** |
| Dyadic CIB | -0.045 | -0.087 – -0.002 | **0.038** | 0.031 | -0.009 – 0.071 | 0.127 |
| Time of day | -0.022 | -0.060 – 0.016 | 0.258 | -0.020 | -0.060 – 0.019 | 0.306 |
| Age | -0.060 | -0.144 – 0.025 | 0.163 | -0.015 | -0.033 – 0.002 | 0.091 |
| Sex | 0.044 | -0.179 – 0.266 | 0.696 |  |  |  |
| Medication yes/no | 0.033 | -0.255 – 0.320 | 0.822 | 0.016 | -0.233 – 0.265 | 0.900 |
| Physical activity | 0.000 | -0.000 – 0.000 | 0.914 | -0.000 | -0.000 – 0.000 | 0.518 |
| BMI | -0.007 | -0.034 – 0.021 | 0.631 | -0.004 | -0.025 – 0.016 | 0.667 |
| Smoking yes/no | -0.155 | -0.389 – 0.080 | 0.192 | -0.166 | -0.392 – 0.060 | 0.147 |
| State:Context3 | -0.199 | -0.481 – 0.083 | 0.167 | -0.212 | -0.460 – 0.035 | 0.093 |
| State:Context4 | 0.103 | -0.124 – 0.329 | 0.373 | 0.059 | -0.121 – 0.239 | 0.519 |
| State:Context5 | 0.050 | -0.208 – 0.308 | 0.703 | 0.069 | -0.150 – 0.287 | 0.537 |
| State rMSSD:Dyadic CIB | -0.007 | -0.165 – 0.151 | 0.933 | -0.017 | -0.138 – 0.104 | 0.781 |
| Context3:Dyadic CIB | 0.023 | -0.024 – 0.069 | 0.337 | -0.048 | -0.092 – -0.004 | **0.033** |
| Context4:Dyadic CIB | 0.005 | -0.032 – 0.041 | 0.797 | -0.025 | -0.059 – 0.008 | 0.137 |
| Context5:Dyadic CIB | -0.007 | -0.051 – 0.036 | 0.743 | -0.052 | -0.093 – -0.010 | **0.014** |
| State:Context3:Dyadic CIB | 0.311 | 0.083 – 0.538 | **0.007** | 0.219 | 0.024 – 0.414 | **0.028** |
| State:Context4:Dyadic CIB | -0.066 | -0.239 – 0.107 | 0.454 | -0.049 | -0.184 – 0.086 | 0.477 |
| State:Context5:Dyadic CIB | 0.124 | -0.064 – 0.312 | 0.195 | 0.180 | 0.014 – 0.346 | **0.033** |
| **Random Effects** | | | | | | |
| Intercept Variance | 0.11 _id_ | | | 0.13 _id_ | | |
| State Variance | 0.06 _id/state_ | | | 0.03 _id/state_ | | |
| ICC | 0.67 | | | 0.73 | | |
| N | 64 _id_ | | | 64 _id_ | | |
| Observations | 1728 | | | 1728 | | |
| Marginal R^2^ / Conditional R^2^ | 0.217 / 0.739 | | | 0.127 / 0.760 | | |

*Note.* Reference category: Context1=Resting 1. Context2=Positive Interaction, Context3 =Resting 2, Context4=Stress Task, Context5=Resting 3. BPD = clinical group; HC = healthy controls.

**Table S3**

*Clinical status and context do not shape vmHRV-Synchrony in randomly assigned mother-adolescent dyads*

|  | **Mother RMSSD -> Adolescent RMSSD** | | | **Adolescent RMSSD -> Mother RMSSD** | | |
| --- | --- | --- | --- | --- | --- | --- |
| *Predictors* | *Estimates* | *Conf. Int. (95%)* | *P-Value* | *Estimates* | *Conf. Int. (95%)* | *P-Value* |
| Intercept | 3.547 | 3.287 – 3.808 | **<0.001** | 3.249 | 3.069 – 3.430 | **<0.001** |
| Time of day | -0.018 | -0.055 – 0.019 | 0.343 | -0.025 | -0.069 – 0.018 | 0.251 |
| Age | -0.052 | -0.131 – 0.027 | 0.190 | -0.010 | -0.027 – 0.008 | 0.284 |
| Medication yes/no | 0.082 | -0.209 – 0.372 | 0.577 | -0.043 | -0.311 – 0.225 | 0.750 |
| Physical activity | 0.000 | -0.000 – 0.000 | 0.976 | -0.000 | -0.000 – 0.000 | 0.381 |
| BMI | 0.003 | -0.024 – 0.031 | 0.811 | 0.000 | -0.023 – 0.023 | 0.979 |
| Smoking yes/no | -0.170 | -0.408 – 0.068 | 0.157 | -0.178 | -0.412 – 0.057 | 0.136 |
| Sex | 0.085 | -0.135 – 0.305 | 0.443 |  |  |  |
| Average rMSSD | 0.149 | -0.072 – 0.370 | 0.182 | 0.088 | -0.186 – 0.361 | 0.525 |
| State rMSSD | 0.172 | -0.021 – 0.366 | 0.081 | 0.112 | -0.031 – 0.256 | 0.123 |
| Group(BPD/HC) | 0.325 | 0.110 – 0.539 | **0.004** | 0.051 | -0.175 – 0.278 | 0.652 |
| Context2 | 0.101 | 0.048 – 0.154 | **<0.001** | 0.112 | 0.064 – 0.161 | **<0.001** |
| Context3 | 0.089 | 0.029 – 0.148 | **0.004** | 0.090 | 0.036 – 0.144 | **0.001** |
| Context4 | 0.061 | 0.008 – 0.114 | **0.024** | 0.111 | 0.063 – 0.160 | **<0.001** |
| Context5 | 0.260 | 0.196 – 0.323 | **<0.001** | 0.237 | 0.177 – 0.297 | **<0.001** |
| State:CG | 0.006 | -0.255 – 0.266 | 0.965 | 0.079 | -0.167 – 0.326 | 0.528 |
| State:Context2 | -0.200 | -0.429 – 0.029 | 0.087 | -0.145 | -0.319 – 0.029 | 0.102 |
| State:Context3 | -0.192 | -0.452 – 0.067 | 0.146 | -0.109 | -0.303 – 0.084 | 0.267 |
| State:Context4 | -0.152 | -0.377 – 0.073 | 0.184 | -0.081 | -0.250 – 0.088 | 0.347 |
| State:Context5 | -0.220 | -0.452 – 0.012 | 0.063 | -0.163 | -0.365 – 0.038 | 0.112 |
| HC:Context2 | -0.102 | -0.180 – -0.024 | **0.010** | -0.055 | -0.126 – 0.017 | 0.133 |
| HC:Context3 | -0.106 | -0.194 – -0.017 | **0.019** | -0.031 | -0.110 – 0.049 | 0.449 |
| HC:Context4 | -0.115 | -0.193 – -0.036 | **0.004** | -0.038 | -0.110 – 0.034 | 0.302 |
| HC:Context5 | -0.092 | -0.186 – 0.001 | 0.052 | -0.021 | -0.111 – 0.068 | 0.638 |
| State:HC:Context2 | 0.034 | -0.286 – 0.354 | 0.834 | -0.079 | -0.371 – 0.213 | 0.597 |
| State:HC:Context3 | -0.055 | -0.426 – 0.317 | 0.773 | -0.153 | -0.467 – 0.160 | 0.337 |
| State:HC:Context4 | -0.040 | -0.361 – 0.281 | 0.808 | -0.117 | -0.416 – 0.181 | 0.441 |
| State:HC:Context5 | -0.185 | -0.531 – 0.160 | 0.292 | -0.282 | -0.622 – 0.058 | 0.104 |
| **Random Effects** | | | | | | |
| Intercept Variance | 0.11 _shuffle_id_ | | | 0.14 _shuffle_id_ | | |
| State Variance | 0.03 _shuffle_id/state_ | | | 0.03 _shuffle_id/state_ | | |
| ICC | 0.63 | | | 0.72 | | |
| N | 64 _shuffle_id_ | | | 64 _shuffle_id_ | | |
| Observations | 2050 | | | 2050 | | |
| Marginal R^2^/Conditional R^2^ | 0.225 / 0.713 | | | 0.107 / 0.752 | | |

*Note.* Reference category: Context1=Resting 1. Context2=Positive Interaction, Context3 =Resting 2, Context4=Stress Task, Context5=Resting 3. BPD = clinical group; HC = healthy controls.

**Figures**

**Figure S1**

Sensitivity Analysis for RQ1

**Note.** Effect sizes of the two-way interaction Context*Group were changed by artificially adding a contribution to the stress task for healthy control (HC). Power was calculated via bootstrapping.

**Figure S2** *Adolescent resting state HRV for different levels of general psychopathology (assessed with the Strength and Difficulties Questionnaire, SDQ).*


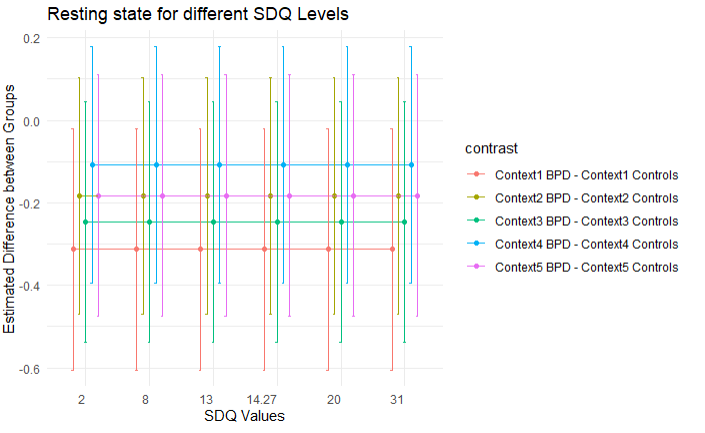


***Note.*** Context 1 = Resting 1, Context 2 = Positive interaction, Context 3 = Resting 2, Context 4 = Stress task, Context 5 = Resting 3. SDQ values correspond to Min, Max, Quartiles, and Mean = 14.27. Results for resting state HRV remain robust for adolescents.

**Figure S3**

*Adolescent HRV reactivity for different levels of general psychopathology (assessed with the Strength and Difficulties Questionnaire, SDQ).*


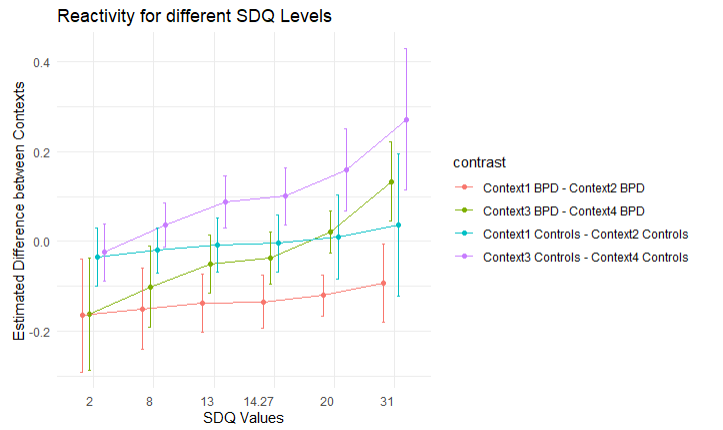


***Note.*** Context 1 = Resting 1, Context 2 = Positive interaction, Context 3 = Resting 2, Context 4 = Stress task, Context 5 = Resting 3. SDQ values correspond to Min, Max, Quartiles, and Mean = 14.27. Results for HRV reactivity remain robust for the difference between Resting 1 to positive interaction. Results for HRV reactivity change for the difference between Resting 2 and the stress task: in both groups, higher SDQ values were associated with higher HRV differences between contexts.

**Figure S4**

*Adolescent HRV recovery for different levels of general psychopathology (assessed with the Strength and Difficulties Questionnaire, SDQ).*


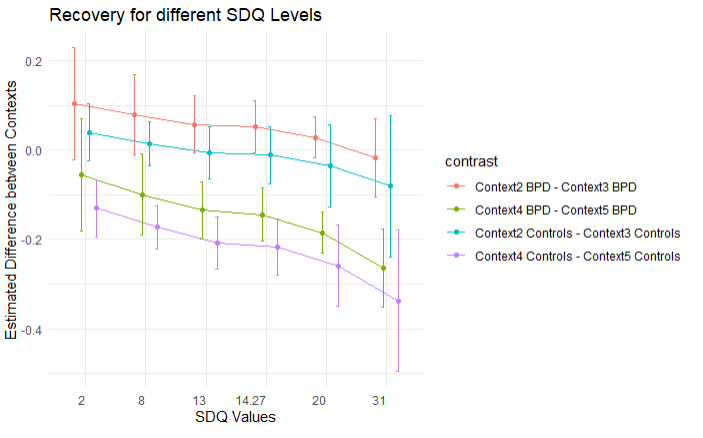


***Note.*** Context 1 = Resting 1, Context 2 = Positive interaction, Context 3 = Resting 2, Context 4 = Stress task, Context 5 = Resting 3. SDQ values correspond to Min, Max, Quartiles, and Mean = 14.27. Results for HRV recovery remain robust for adolescents.

**Figure S5**

*Intrapersonal concordance of HRV and behavior***.**

*Note.* Context 3, 5=Resting: Context 2=Positive interaction, Context 4=Stress task. The adolescent model was not significant; in the maternal model a significant positive HRV-behavior association during the positive interaction was found.

**Figure S6**

Sensitivity Analysis for RQ3

**Notes.** Effect sizes of the three-way interaction Context*Group*state rMSSD were changed by artificially adding a dependency on state rMSSD to the positive interactionstress task for healthy control (HC). Power was calculated via bootstrapping.

**Figure S7**

*Dyadic CIB and context shape vmHRV synchrony.*

*Note.* Context 3, 5=Resting: Context 2=Positive interaction, Context 4=Stress task. When behavioral synchrony is lower, significant positive vmHRV-Synchrony during stress task in both models. When behavioral synchrony is average, significant state vmHRV associations during resting after stress (Context 5) in both models. When behavioral synchrony is higher, significant vmHRV-Synchrony during resting after stress and after positive interaction in both models (Context 3).
